# Empowerment of patients in online discussions about medicine use

**DOI:** 10.1186/s12911-015-0146-6

**Published:** 2015-04-08

**Authors:** Jasper J van Berkel, Mattijs S Lambooij, Ingrid Hegger

**Affiliations:** Dutch National Institute for Public Health and the Environment (RIVM), PO Box 1, NL-3720 BA Bilthoven, The Netherlands

**Keywords:** Internet, Online support groups, Social media, Online forum, Empowerment, ADHD, ALS, Diabetes

## Abstract

**Background:**

Patient empowerment is crucial in the successful self-management of people with chronic diseases. In this study, we investigated whether discussions about medicine use taking place on online message boards contribute to patient empowerment and could subsequently result in the more effective use of medicines. We discuss the extent to which patient empowerment processes occur in discussions on online message boards, focusing on patients with three disorders with different characteristics: diabetes, Amyotrophic Lateral Sclerosis (ALS) and Attention Deficit / Hyperactivity Disorder (ADHD). Because information is an important factor in both patient empowerment and self-management, we also evaluate the quality of the information being exchanged.

**Methods:**

We used a deductive thematic analysis method based on pre-existing categories. We gathered and analysed 5532 posts related to the conditions ADHD, ALS and diabetes from seven message boards (three for ADHD, three for diabetes, and one for ALS). We coded the posts for empowerment processes and the quality of the information exchanged.

**Results:**

We identified patient empowerment processes in posts related to all three disorders. There is some variation in the frequency of these processes, but they show a similar order in the results: patients used the online message boards to exchange information, share personal experiences and for empathy or support. The type of information shared in these processes could contribute to the patient’s self-efficacy when it comes to medicine use. The exchanged information was either correct or largely harmless. We also observed a tendency whereby participants correct previously posted incorrect information, and refer people to a healthcare professional following a request for medical advice, e.g. concerning the choice of medicines or dosage.

**Conclusions:**

Our findings show that patient empowerment processes occur in posts related to all three disorders. The type of information shared in these processes can contribute to the patient’s self-efficacy when it comes to medicine use. The tendency to refer people to a healthcare professional shows that patients still reserve an important role for healthcare professionals in the care process, despite the development towards more self-management.

**Electronic supplementary material:**

The online version of this article (doi:10.1186/s12911-015-0146-6) contains supplementary material, which is available to authorized users.

## Background

In recent years, patients with chronic conditions are expected to be more self-sufficient in the management of their disorder; they are expected to take control, take responsibility, and manage their own care process as far as possible [1,2]. This places high demands on patients, as they need to be aware of what their condition involves, what the consequences are, and which actions they can take to prevent complications [3]. It is estimated that around 90% of people with a chronic disease are prescribed medicines for long-term use [4,5], with self-management being sufficient for 70 to 80% of patients [3]. Self-management of medicines is important, as the response to treatment largely depends on the medicine schedule and the dosage. However, low adherence to prescribed treatment is very common in patients. It is estimated that typical adherence rates for prescribed medicines are as low as 50% [6]. With the increasing call for self-management, it becomes more and more important for patients to gain experience in managing their medicines.

Previous research has shown that when people take an active role in managing their health and increase their self-efficacy, this contributes to the successful implementation of self-management [7,8]. Both health knowledge and empowerment have been shown to have a major impact on the self-management of chronic conditions [9]. The concept of empowerment has been gaining in popularity in recent years [10]. For example, patient empowerment has been shown to improve blood glucose control in diabetes patients [11]. Other benefits of empowerment for patients include enhanced social well-being, increased self-efficacy, becoming better informed, improved confidence in treatment, improved acceptance of their illness, and feeling more competent and in control [12-16]. At the heart of patient empowerment lies the assumption that “patients are experts on their own bodies, symptoms and situation, and this knowledge is necessary to succeed in treatment” [1]. This approach regards the patient as a partner in healthcare, with both the patient and the healthcare professional having their own rights and responsibilities. This changes the hierarchical relationship between doctor and patient. By becoming better informed, patients can play a more active role in consultations and decision-making. This therefore implies that healthcare is “moving away from the traditional asymmetric power balance inherent in the medical model” [12], with the patient being dependent on the healthcare professional. “Empowering” patients reduces the traditional information asymmetry. Patients can obtain medical information through interaction with a doctor, but increasingly also via the Internet.

Since information is a key element in patient empowerment and the Internet is an endless source of information, the promotion of patient empowerment is likely to be influenced by the information-seeking behaviour of patients on the Internet. Research has shown that the Internet is increasingly used by people to search autonomously for health-related information [17,18], creating what Fox [19] calls “peer-to-peer healthcare”. Patients gather information before going to a healthcare provider in order to determine whether a visit is necessary. After their visit, patients look for more detailed or reassuring information [20]. While this is an example of self-efficacy and therefore a potentially important source of patient empowerment, poor-quality information found online can lead to possible adverse effects [21-23]. In addition, there are concerns about the dangers of using the Internet to obtain medicines outside the official medical system [24]. This problem may be solved in part through website accreditation by professionals or healthcare professionals assuming an active facilitating role to help patients assess the value of online information [22,23,25]. In the case of online resources such as message boards, however, information of good quality is much harder to achieve or to guarantee, as the information is dependent on a large number of often anonymous individuals. Other potential dangers or disempowering effects of using online message boards include negative posts, disadvantages related to the use and evaluation of healthcare services, asynchronous communication, anonymity, lack of physical contact, and the large amount of information generated on message boards [26-28]. Despite these possible disadvantages, studies have shown that patients feel empowered when using social media [12,14,18,26,29].

Therefore, in this study we examine whether empowerment processes occur on message boards discussing medicines used to treat three chronic diseases: diabetes (Type 1 and 2), ADHD and ALS. Because information plays an important role in both empowerment and successful self-management, we also evaluated the quality of information about medicine use that is exchanged on online message boards between patients suffering from these diseases.

## Methods

### Sample and procedure

We selected the aforementioned three disorders because of their different characteristics, since we expected to find different empowerment mechanisms for different diseases. Lifestyle plays an important role in ADHD and diabetes. One difference between these two conditions is that there is a public debate about whether or not ADHD is an actual disorder [30], while diabetes is accepted as a disorder. In the case of ADHD, empowerment means being diagnosed and accepted as someone who has ADHD, in order to get access to medicines (without prejudice). In contrast, empowerment in the case of diabetes is focused on successfully managing the condition in daily life, with the use of insulin playing an important role. The rare and fatal disease ALS differs from the other two disorders because there are hardly any therapeutic options available [31-33]. In the case of ALS, empowerment means being well-informed as a patient and getting access to new (experimental) medicines.

For each disorder, we used Google to find those Dutch message boards where relevant medicines were most frequently discussed. The query included the disorder name, names of relevant medicines, and typical extensions of (Dutch) message boards (see Additional file 1). The search was limited to Dutch websites and a period of five years (July 2008 – July 2013). This resulted in a selection of 70 message boards (27 for ADHD, 35 for diabetes and 8 for ALS; see Additional file 2). Using Google, we searched each message board for discussions about the medicines used to treat the three disorders. This provided us with an overview of the most active message boards for each disorder (again limited to a period of five years). We limited our search to publicly accessible message boards, since it is likely that any Dutch patient looking for information and using similar keywords when searching for information would also end up on one of these websites. We repeated the search daily during a one-week period to account for any deviations in the search results.

For further analysis, we selected the three most active message boards for each disorder, unless they contained content not related to the human disorder. For example, one message board containing information about horses with diabetes was not included, but did end up in the search results because of the keywords ‘diabetes’ and ‘insulin’. In total, we selected seven message boards for further analysis (three for ADHD, three for diabetes, one for ALS). The two most active message boards for both ADHD and diabetes were Fok and Viva. The VIVA message board is primarily aimed at adult women, whereas the FOK message board is primarily aimed at young people. As a result, different norms apply on the two message boards. The VIVA message board tends to be more supportive in nature, while discussions on the FOK message board tend to be more challenging. Both message boards are general in nature, which means that they target a broad audience and not just patients. The third most active ADHD message board is Babybrabbel, which is aimed at women who are pregnant or recently had a baby. Babybrabbel is similar to VIVA in the sense that it is a general message board where the discussions and exchanges are supportive in nature. Diabetesforum was the final message board about diabetes that was selected. This forum is specifically aimed at diabetes patients, and many of the posts are about exchanging experiences of living with this condition. Due to the rareness of ALS, only the ALS-specific message board StopALS.nu contained posts related to the use of ALS medicines. This message board is aimed at patients suffering from ALS and those close to them. Moderators were present on all forums, but mainly visible in the discussions about ADHD on the general message boards. From the selected message boards, we downloaded (in the form of a PDF file) every thread that included posts about relevant medicines; this resulted in 501 downloaded threads. All the threads were loaded using the ATLAS.ti program. For every disorder, we selected the first 25 threads on each message board. We excluded threads if they contained only a very small number of posts discussing medicine use. We analysed the individual posts within the context of the thread. For two message boards, less than 25 threads related to medicine use were available (see Additional file 2). In total, we coded 5532 posts (2517 on ADHD, 2467 on diabetes, and 548 on ALS).

The posts were coded using a deductive thematic analysis method. This type of analysis is useful in research aimed at answering a specific research question, for the purpose of identifying, analysing and reporting themes or patterns within data [34,35]. In our study we focused on the type of empowerment processes that occur in online forums. First we familiarised ourselves with the data and read all the threads and we wrote down our initial ideas. We found that these corresponded with the processes described by van Uden-Kraan, et al. [14,26] and therefore considered these ideas well-suited as a coding framework for this study. The first author proceeded to code each post. The coding was not based on verbal cues; instead posts (or fragments of posts) were placed in the predefined coding scheme. To validate the coding, the second and third author checked a random sample of posts in an open coding session to obtain agreement on the coding. The final analysis was performed based on a consensus reached between all three authors. To provide an indication of the relative prevalence of the different empowerment processes, we have also specified how many times these processes occur in the posts.

After the first coding session, the third author, being a pharmacist by education, evaluated the posts that provided information on medicines and their use. The focus was on assessing whether the information might have harmful effects. The categories were not based on previous research, but emerged from the type of information provided in the posts. For example, advice on discontinuing the use of medicines or changing the dose without consulting a doctor was considered to be poor advice. Advice we considered to be of high quality included the recommendation to consult a medical professional when considering changing medicine intake or use.

It was possible to assign multiple codes to a post, as different subjects could be covered in the same post. Posts depend on the threads they are part of, because many of them contain a response to previous posts. Hence, we coded the posts as such based on earlier work by Finn [36] and van Uden-Kraan, et al. [26].

To protect the privacy of the message board users, the quotes used in this article were translated from Dutch to English to ensure they cannot be traced back to the original source. For the analysis, we used the original text. According to the Dutch National Ethics Board (Central Committee on Research Involving Human Subjects), formal review by a medical ethics committee was not necessary, as the people involved were not subject to treatment or required to follow a certain behavioural strategy. This is in accordance with the guidelines laid down in the Declaration of Helsinki. By limiting our study to publicly available anonymous information, the study is in accordance with Dutch privacy and data protection legislation.

### Measures

#### Empowerment processes

Empowerment processes have been discussed in several articles. Finn [36] has described the following processes occurring in an online self-help group about disability: *mutual problem solving, information sharing, expression of feelings, catharsis, mutual support and empathy*. Building on this research, Perron [37] described the following empowerment processes: *disclosure, providing information or advice, empathy or support, gratitude, requesting information or advice, computer issues, friendship, creative expression, structure*. van Uden-Kraan, et al. [26] confirmed the occurrence of these processes in online forums. They also found the following empowerment processes in online support groups about breast cancer, arthritis and fibromyalgia: *exchanging information, encountering emotional support, finding recognition and understanding, sharing experiences and helping others, amusement* [14]. Several studies [12,27,38] have confirmed the occurrence of these empowerment processes and also confirmed the outcomes described by van Uden-Kraan, et al. [14]. Two other studies into social support for weight loss in online communities show similar results [39,40]. The major social support themes found were: *encouragement and motivation, information, and shared experiences*. These themes are closely related to the processes found by van Uden-Kraan, et al. [14] [15]. An online survey conducted by Holbrey, et al. [41] among 50 participants revealed several other empowerment processes: *connecting with others who understand, access to information and advice, interaction with healthcare professionals, treatment-related decision-making, improved adjustment and management*.

We chose to use a categorization based on the work of van Uden-Kraan, et al. [14,26] because they not only described empowerment processes in online forums, but also described positive empowerment outcomes related to these processes. This resulted in the following categories in our coding scheme: *providing information, requesting information, sharing personal experiences, exchanging empathy or support, gratitude* and *comparison with other members*. A separate category was added for posts that contain *off-topic, everyday talk* [26].

## Results

### Empowerment processes

Table 1 provides an overview of the frequency of occurrence of the empowerment processes in the analysed threads. The two most prominent empowerment processes identified were *providing information* and *sharing personal experiences.*

**Table 1:** **Frequency of occurrence of patient empowerment processes**

**Empowerment process**	**ADHD**		**Diabetes**		**ALS**		**Total**	
	(n = 2517)*		(n = 2467)*		(n = 548)*		(n = 5532)*	
	**n**	%**	**n**	%**	**n**	%**	**n**	%**
Providing information	1114	44.0	1130	46.0	294	53.5	2538	46.0
Sharing personal experiences	912	36.0	1152	47.0	251	46.0	2315	42.0
Requesting information	357	14.0	472	19.0	127	23.0	956	17.0
Exchanging empathy or support	146	6.0	324	13.0	54	10.0	524	9.5
Gratitude	39	1.5	111	4.5	16	3.0	166	3.0
Comparison with other members	57	2.0	74	3.0	9	1.5	140	2.5

*Number of posts **Not cumulative (multiple topics can occur in a post).

Table 1 shows that *providing information* is the most frequent activity. This is because most posts in this category are responses to information requests which triggered more than one post providing information per request. We also found that sharing *personal experiences* is an important activity on online message boards. These posts often provide information as well, as personal experiences are used to illustrate possible choices regarding medicine use. In order to find out which healthcare-related information people seem not to possess, we will focus on the information requests made by patients*.*

In contrast to previous findings [26], we classified a relatively small number of posts as *off-topic* (less than 5% of the total number of posts). We found most of the off-topic posts on the general message boards, with no significant difference between the disorders. We believe this is the result of the search criteria used in this study, which resulted in threads where medicines are mentioned at least once.

Table 2 provides an overview of the most frequently occurring topics for each disorder, based on the information requested. Almost half of all questions concerned requests for *supplementary information* in reply to an earlier statement made by a user. *Effects of medicines* were most frequently discussed in the posts about ADHD and ALS; these mainly concerned the experiences of other people using the relevant medicines. In the ADHD-related posts, people occasionally discussed side-effects, whereas side-effects played only a minor role in discussions about ALS and diabetes. The same applies to the topic *options of medicines*, which was primarily discussed in posts concerning ADHD. In these cases, people asked for information about which medicines to take or about potential alternatives: *“I’m looking for an alternative medicine to treat ADHD/ADD. I have tried Ritalin, Concerta and other medicines, but I have experienced some side-effects. When the side-effects wear off, I get depressed, a bit like a hangover”.*

**Table 2:** **Topics on which information is requested**

**Topics**	**ADHD**		**Diabetes**		**ALS**		**Total**	
	(n = 357)*		(n = 472)*		(n = 127)*		(n = 956)*	
	**n**	%**	**n**	%**	**n**	%**	**N**	%**
Supplementary information	134	37.5	283	60.0	57	45.0	474	49.5
Effects of medicines	70	19.5	4	1.0	20	16.0	94	10.0
Options of medicines	45	12.5	-	-	3	2.5	48	5.0
Dealing with medicines/disorder	17	5.0	24	5.0	8	6.5	49	5.0
Availability of medicines	34	9.5	-	-	7	5.5	41	4.5
Use of medicines	13	3.5	17	3.5	8	6.5	38	4.0
Dosage	14	4.0	17	3.5	2	1.5	33	3.5
Blood sugar levels	-	-	27	5.5	-	-	27	3.0
Diagnosis	-	-	22	4.5	-	-	22	2.5
Trial studies	-	-	-	-	8	6.5	8	1.0
Other	30	8.5	78	17	14	10	122	13.0

*Number of posts in which information is requested **Percentages have been rounded to one decimal.

Questions about *dealing with medicines or the disorder itself* were mostly about the integration of medicines into daily activities: *“You mentioned that you are currently not using any medicines. How do you cope with this at work?”* Besides this question of how people deal with medicines at work, users also asked questions about the use of medicines during vacations or at parties. The ALS questions were more about the disorder itself, as they focused on practicalities like home modifications to ensure that patients can live independently for longer.

Only the ADHD and ALS posts discussed the *availability of medicines*. For ADHD, the emphasis was on how to obtain approved medicines, whereas the emphasis in the ALS posts was on obtaining access to experimental medicines in clinical development: *“Will non-participants [in the trial] also receive Dexpramipexol in October and how do I obtain access to this medicine? Do I need a prescription from the doctor or neurologist? I’m curious!”* In addition to Dexpramipexol, there were many questions about how to obtain the supplements used in the so-called ‘Deanna protocol’, which is an experimental treatment based on food supplements. In the case of ADHD, people asked questions about the insurance coverage of specific medicines, and the costs of obtaining a diagnosis from a doctor (in order to gain access to medicines).

We found several posts in which people were looking for a *diagnosis* on whether they had diabetes: *“Maybe one of you has experienced this as well, where just the fact that you drink a lot was an indication of diabetes?”* Other posts discussed how a diagnosis could be made, or whether you need to be sober when visiting the doctor for a diagnosis.

Some questions could have (negative) medical consequences, for instance those about topics like *medicine options*, *dosage* or *diagnosis*. However, users who requested medical information were often referred to a healthcare professional. An example of this was when a user inquired about an alternative to methylphenidate: *“There are other types of medicines besides methylphenidate, perhaps Strattera is better suited for you. Discuss this with your psychiatrist and try to find an alternative”.* People were also advised to visit a doctor to obtain a diabetes diagnosis, and were advised not to adjust their dosage without consulting a doctor.

### Quality of the information

The quality of the information in posts was assessed using the four categories shown in Table 3. The source or substantiation of the information provided was generally unclear, although a small number of posts referred to a website, scientific article or advice from a healthcare professional. The majority of the posts contained harmless information, meaning that the information presented does not pose a risk to the reader. One example of this is a reply to a question about whether it is still safe to use insulin that has been temporarily subjected to higher temperatures than recommended: *“When in doubt, just throw it away. Feeling unwell as a result of spoiled insulin is not worth the cost of a new vial”.*

**Table 3:** **Quality of information provided in posts**

**Quality of information**	**ADHD**		**Diabetes**		**ALS**		**Total**	
	(n = 1114)*		(n = 1130)*		(n = 294)*		(n =2538)*	
	**N**	%**	**n**	%**	**n**	%**	**n**	%**
Harmless information	712	64.0	853	75.5	147	50,0	1712	67.5
Correct information	190	17.0	190	17.0	6	2,0	386	15.0
Disputable information	157	14,0	80	7.0	144	49,0	381	15.0
Incorrect information	61	5.5	8	0.5	-	-	69	2.5

*Number of posts providing information **Not cumulative (some posts contained multiple codes).

The second most frequently occurring category consisted of posts containing *correct information* or *disputable information*. An example of correct information is the advice to visit a doctor. This advice was found in almost every thread where a person was looking for specific medical information: *“Instead of experimenting with medicines on your own, it might be a good idea to go to a doctor”.* Other examples include people being referred to the patient information leaflet or a recommendation not to break tablets in half. Posts containing disputable information included advice that could have adverse effects when followed. Common examples of this were posts advising or informing the reader to start using different medicines: *“Dexamphetamine is garbage; ask for a prescription for methamphetamine”.* Other posts advised users to increase or reduce their dosage or to stop taking medicines altogether.

Finally, a small number of posts contained *incorrect information*. This concerned information that was clearly incorrect, with a high risk of adverse effects. One example concerns a post recommending the recreational use of Ritalin: *“This has been happening for years – it’s relatively harmless… have fun :)”* Some posts offered tips on how to break tablets in order to obtain the right dose, which could potentially lead to a dose that is either too high or too low: *“I cut them in half, perhaps a tip?”* In a few cases, people shared the fact that they acquired medicines without a prescription outside the official medical system. They stated they obtained medicines from friends or through an online auction site, but did not provide further specifics: *“Received Ritalin from a friend – it seems like I have more focus, so it’s fine!”* Many posts that contained disputable or incorrect information were responded to by other users who either corrected the information or warned people of the possible dangers.

## Discussion

We found some variation in the frequency of empowerment processes for the different disorders. This may be partly explained by the nature of the different disorders. Both van Uden-Kraan, et al. [14] and our study show that the smallest number of empowerment processes occurred on message boards about controversial disorders. In the case of ADHD, there is a lack of consensus concerning the nature and treatment of the disorder [30]. We found that discussions about the validity of the disorder occurred in many threads. This could have a negative impact on the empowerment process of the users participating. Future research could determine (i) whether this trend also plays a role in other controversial diseases; and (ii) the extent to which this affects a patient’s empowerment process.

In line with earlier studies [12,26], our results show that providing information and sharing personal experiences are the most frequently occurring empowerment processes. In contrast to other studies, the third most prominent process was requesting information. The category ‘providing empathy or support’ was less prominent than in other studies, where this category occurred as frequently as providing information and sharing personal experiences [12,26]. One possible explanation is that more people participate in general message boards who are not affected by the disorder and who have less incentive to contribute to empowerment processes such as providing empathy or support. However, this does not explain the lower rate of occurrence on the ALS message board, which is primarily aimed at patients and relatives.

Online platforms have become increasingly popular sources for gathering information about patients sharing their medicine use experiences [42], including the possibility to detect possible side-effects at an earlier stage [43]. In our study users seemed to focus mainly on whether or not medicines had the intended effect. Discussions about side-effects were mostly limited to the ADHD message boards. The type of information exchanged about the use of medicines could help increase the self-efficacy of patients, enabling users to receive information more quickly and tailored to their personal needs [14]. We found that people were actively exchanging information about medicine use, either by providing information, sharing personal experiences, or requesting information. The most prominent topics discussed concerned the effects of medicines, dealing with medicines or the disorder itself and the use and dosage of medicines. The topics discussed do not necessarily promote patient empowerment, as the posts may contain harmless, correct, disputable or incorrect information. Posts containing disputable or incorrect information could have potentially adverse and disempowering effects when that information is acted upon.

Most of the information provided in the posts was either harmless or correct. We did find a few messages that included disputable or incorrect information. This percentage was considerably higher on the ALS message board, due to a thread discussing a regimen of food supplements. Much of the information discussed in this thread could be considered disputable, as this regimen has not been tested yet. In general, we found that many people respond to and correct posts that include incorrect or disputable information, for example by referring users to a doctor. However, it should be noted that message board moderators may delete disputable information. Although we found no clear indications that such removals occurred in the threads we analysed, our findings may be biased due to the possible deletion of posts. We chose to focus on the quality of information that can be consulted on the Internet at the end. Nevertheless, further research on the effect of moderators on empowerment processes could generate useful information for the strategic application of message boards in healthcare. On the ALS message board we found no posts referring people to a doctor. One possible explanation is that doctors cannot do much to help a patient once the ALS diagnosis has been made, quickly considering the patient as being finished with treatment. However, doing nothing is not an option for many ALS patients due to the fatal outcome of this disease. Many place their hope in other patients’ knowledge of treatment options.

Although much of the information itself may be considered harmless, this does not necessarily mean that the effects cannot be harmful, as the utility of health information depends on the recipient’s background knowledge that is necessary to evaluate the information adequately [44]. The extent to which people rely for information on other people’s experiences as posted on the Internet is a cause of concern as well [45]. Our study reflects the importance of personal experiences, but we found no indications that these posed more risk than other types of information.

Previous research has shown that patient empowerment enhances a patient’s decision-making in treatment [11]. As this study did not survey the users involved, we cannot verify to what extent this actually occurred. However, patients active on the Internet seem to be knowledge-acquirers rather than decision-makers [13,18]. Previous research has shown that “3% of patients changed their medication without consulting a healthcare professional and 7% made/cancelled/changed a consultation as a result of information from the Internet” [18]. This corresponds to our finding that people are often referred to healthcare professionals to make decisions related to their treatment. Wentzer, et al. [15] noted that this could bolster adherence, as the dominant narrative on a message board leaves little room for reflection. In our study, the dominant view on the message boards seemed to favour collaboration with a healthcare professional.

Our study was subject to some limitations. Firstly, we focused on Dutch message boards because we intended to conduct the search from a Dutch perspective. It is likely that Dutch patients will also visit English message boards, as most people in the Netherlands are able to read and write English. People confronted with a rare disease such as ALS are probably more inclined to visit international message boards than patients suffering from a common disorder. Therefore, our search parameters may have excluded some message boards, and also resulted in only one relevant ALS message board to use in this study. Secondly, in our study we used a mix of general message boards and message boards about a specific disorder. This might influence the frequency of the empowerment processes found, although we found a similar order of the processes in both types of message boards. Thirdly, when searches are performed on the same computer, Google offers a consistent way to search all message boards in the same manner. At the same time, we found that Google only displays a specific percentage of its total hits. Because of this, we may have missed some posts. Fourthly, moderators deleted a few posts where users offered to sell medicines, and gave the user a warning. We found no clear indications that any other actions were taken by moderators; for example, we saw no “edits” in posts. We also found no responses to deleted posts, e.g. quotes from deleted posts. Nevertheless, there is a possibility that our findings could be biased due to the possible removal of posts. Fifthly, we selected the first 25 threads shown in the ATLAS.ti program after randomly loading the corpus of files. After coding the selected 25 threads, we concluded that the same patterns occurred in the threads. We therefore do not expect that we have missed information.

## Conclusions

This study explored the extent to which aspects of patient empowerment, through patient-to-patient interactions about medicine, are found in discussions on online message boards for diabetes, ALS and ADHD patients. We found that empowerment processes occurred in all the threads we analysed. These processes occurred more frequently in the threads related to diabetes and ALS than in the threads related to ADHD. The three most frequently occurring empowerment processes were *providing information, sharing personal experiences* and *requesting information*.

We found that people actively engage in acquiring information about their medicines. The most prominent topics discussed concerned the effects, options of medicines, dealing with medicines or the disorder itself, and the use and dosage of medicines. Since providing and requesting information were among the most frequently occurring empowerment processes, the absence of control of the quality of information could be a disadvantage. The information provided was often based on personal experiences. The source of information not based on personal experience was generally unclear. We found that the majority of posts contained harmless information, with a low risk of harm. Posts containing disputable information comprised the second most frequently occurring category, which accounted for approx. 15% of the total number of posts. A small number of posts contained incorrect information that could be potentially harmful. However, we also found that people were often referred to a medical professional when they requested advice concerning their personal medical situation.

Our findings show that patient empowerment processes occur in posts related to all three disorders. There is some variation in the frequency of these processes, but they show a similar order in the results. The type of information shared in these processes may contribute to the patient’s self-efficacy when it comes to medicine use. Still, the tendency to refer people to a healthcare professional shows that patients still reserve an important role for healthcare professionals in the care process, despite the development towards more self-management.

## Additional files

Additional file 1:
**Search queries.** Overview of the search queries used in this study. Additional file 2:
**Search results Google.** Overview of the selected message boards in this study.
