# Supplementary material for: Empowerment of patients in online discussions about medicine use
Source: BMC Med Inform Decis Mak. 2015 Apr 8;15:24. doi: 10.1186/s12911-015-0146-6 (PMC4397724; doi:10.1186/s12911-015-0146-6)
Supplement: Additional file 1: — Search queries. Overview of the search queries used in this study. [file 12911_2015_146_MOESM1_ESM.pdf]

Additional file 1 Search queries

ADHD
ADHD ($forum.nl OR /forum OR $forum.$) (Ritalin OR Concerta OR Medikinet OR Eqausym OR Dexamfetamine OR Methylfenidaat OR Atomoxetine OR Lisdexamfetamine OR Clonidine OR Imipramine OR Nortriptyline OR Medikinet OR Aderol OR Aderal OR Aderall)

Diabetes
Diabetes ($forum.nl OR /forum OR $forum.$) (Insuline OR Novorapid OR Novomis OR Levemir OR Lantus OR Apidra OR Humalog OR Actrapid OR Humuline OR Insuman OR Acrtraphane OR Huniline OR Mixtard OR Insulatard)

ALS
"Amyotrofe laterale sclerose" ($forum.nl OR /forum OR $forum.$) ("IGF-I" OR Rilutek OR Glentek OR Riluzol OR Xaliproden OR Arimoclomol OR Dexpramipexole OR Thalidomide OR Lenalidomide)
