# Supplementary material for: Empowerment of patients in online discussions about medicine use
Source: BMC Med Inform Decis Mak. 2015 Apr 8;15:24. doi: 10.1186/s12911-015-0146-6 (PMC4397724; doi:10.1186/s12911-015-0146-6)
Supplement: Additional file 2: — Search results Google. Overview of the selected message boards in this study. [file 12911_2015_146_MOESM2_ESM.pdf]

**Additional file 2 Search results Google**

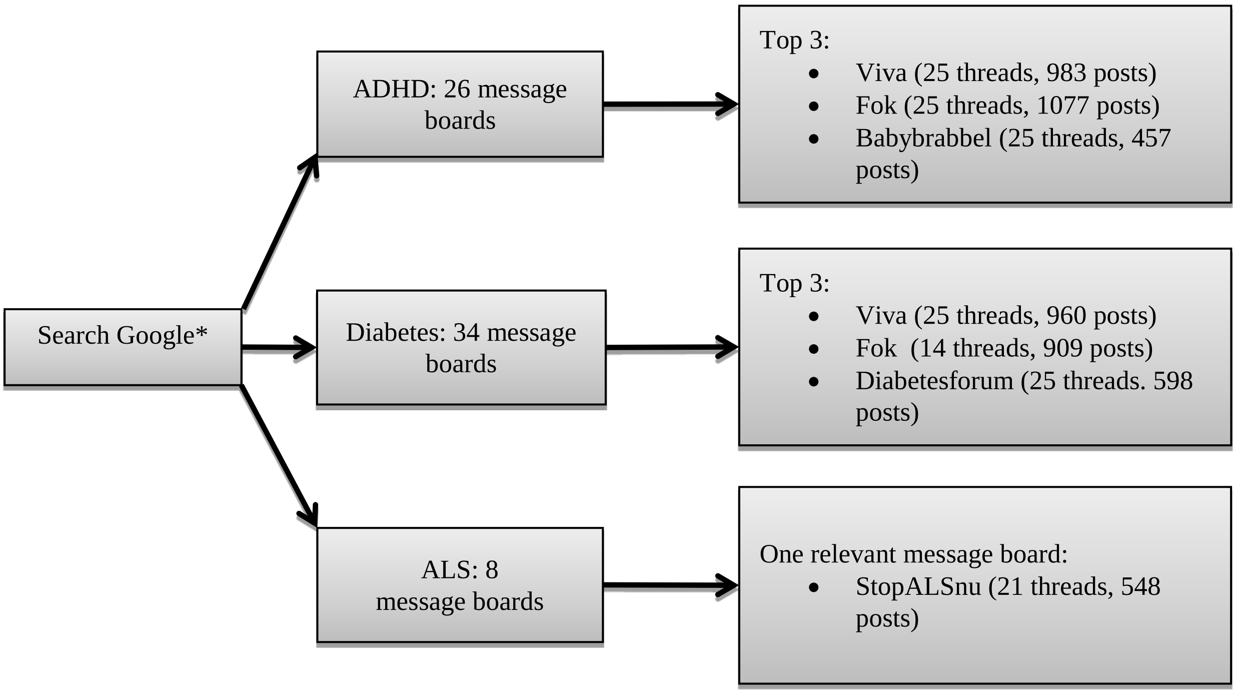


*To get an overview of the most active message boards we performed the same search daily for one week and used an average of the search results. This way we minimized the influence of fluctuations in the search results.
